# Double ileo-ileal intussusception secondary to inflammatory pseudotumor in an adult: A rare case report

**DOI:** 10.1016/j.ijscr.2025.111500

**Published:** 2025-06-11

**Authors:** Sarthak Satpathy, Sibaprashad Pattanayak, Jitendra Kumar Sahoo, Satyaswarup Patnaik, Umesh Chandra Behera, Chinmay Samantaray

**Affiliations:** Department of General Surgery, MKCG Medical College and Hospital, Berhampur, Odisha, India

**Keywords:** Adult intussusception, Inflammatory pseudotumor, Ileum, Small bowel obstruction, Intussusception, Case report

## Abstract

**Introduction:**

Adult intussusception is rare and typically associated with an underlying lead point. Inflammatory pseudotumor, a rare benign lesion of uncertain etiology, can serve as such a lead point in the small bowel. The occurrence of double intussusception in this context is exceptionally uncommon.

**Presentation of case:**

A 55-year-old woman presented with signs of small bowel obstruction. Imaging(Fig. 1–11) revealed ileo-ileal intussusception. Intraoperatively(Fig. 16–18), a Double intussusception configuration was observed where one intussuscepted segment acted as the lead point for a second. A firm lesion at the apex was resected. Histopathology confirmed Inflammatory Pseudotumor(Fig. 12–15). The patient recovered uneventfully and remained asymptomatic at 6-month follow-up.

**Discussion:**

Double intussusception is extremely rare in adults. In this case, an inflammatory pseudotumor triggered an initial ileo-ileal intussusception, which subsequently served as the lead point for a second. The unusual etiology and configuration can pose diagnostic challenges, as radiological imaging may not fully reveal the complexity. Surgical intervention was required for definitive management.

**Conclusion:**

Double ileo-ileal intussusception caused by an inflammatory pseudotumor represents an exceedingly rare etiology of acute intestinal obstruction in adults. Prompt surgical treatment is critical. This case highlights the importance of considering rare benign tumors as potential lead points in adult intussusception.

## Highlights

• Adult intussusception is rare and typically has a pathological lead point.
• Inflammatory pseudotumor is an unusual benign tumor that can cause intussusception.
• This case revealed a unique Double intussusception configuration.
• Prompt surgery enabled both definitive diagnosis and curative treatment.
• Surgeons and radiologists should be aware of such rare presentations.

## 1 Introduction

Intussusception in adults is a rare occurrence, accounting for only 1–5 % of all cases of intestinal obstruction. In contrast to pediatric cases, which are often idiopathic, adult intussusception is typically associated with a pathological lead point, such as neoplasms, Meckel's diverticulum, or postoperative adhesions [1,2]. Inflammatory pseudotumor, a benign neoplasm composed of inflammatory cells and fibroblasts, is a rare but recognized cause of intussusception, particularly in the small intestine [3,4]. Although more commonly found in children and young adults, inflammatory pseudotumors can present in adults as lead points for adult intussusception [5,6].

Double intussusception, where one intussuscepted segment becomes the lead point for another, is exceptionally rare, with only a few cases documented in the literature [7]. These cases often involve benign etiologies, such as inflammatory pseudotumors. The pathophysiology behind double intussusception involves the telescoping of an already intussuscepted segment into another segment, leading to a more complex form of bowel obstruction. Radiological findings and the complexity of the condition require high clinical suspicion and timely surgical intervention.

This report presents a rare case of adult double ileo-ileal intussusception caused by an inflammatory pseudotumor, which was managed successfully at Government Hospital in Berhampur, Odisha, India.

### 1.1 Methods

This case report has been reported in line with the SCARE checklist. [7]

## 2 Presentation of case

A 55-year-old woman was brought by ambulance to the emergency department with progressively worsening abdominal pain for three days, accompanied by nausea, multiple episodes of bilious vomiting, abdominal distension, and absence of bowel movements or flatus. She denied any prior similar episodes, weight loss, or gastrointestinal bleeding. Her past medical and surgical histories were unremarkable.

On examination, the patient's vital signs were stable. The abdomen was distended with localized tenderness in the periumbilical and right lower quadrant regions. There was no guarding or rebound tenderness. Bowel sounds were hypoactive.

The patient presented with signs of small bowel obstruction, including bilious vomiting, abdominal distension, and absence of bowel movements or flatus. Initial management included intravenous fluids, nasogastric decompression, antiemetics, and broad-spectrum antibiotics due to concerns about bowel ischemia.

Laboratory investigations showed a White Blood Cell count of 12,800/mm^3^, hemoglobin of 13.4 g/dL, and an elevated C-reactive protein level of 40 mg/L. Electrolytes and renal function were normal.

### 2.1 Imaging

A non-contrast computed tomography (CT) scan revealed features suggestive of ileo-ileal intussusception. The radiologist noted a possible lead point lesion, with no evidence of perforation or ischemia; however, a double intussusception configuration was not suspected preoperatively based on imaging.(Fig. 1, Fig. 2, Fig. 3, Fig. 4, Fig. 5, Fig. 6, Fig. 7, Fig. 8, Fig. 9, Fig. 10).

**Fig. 1:**
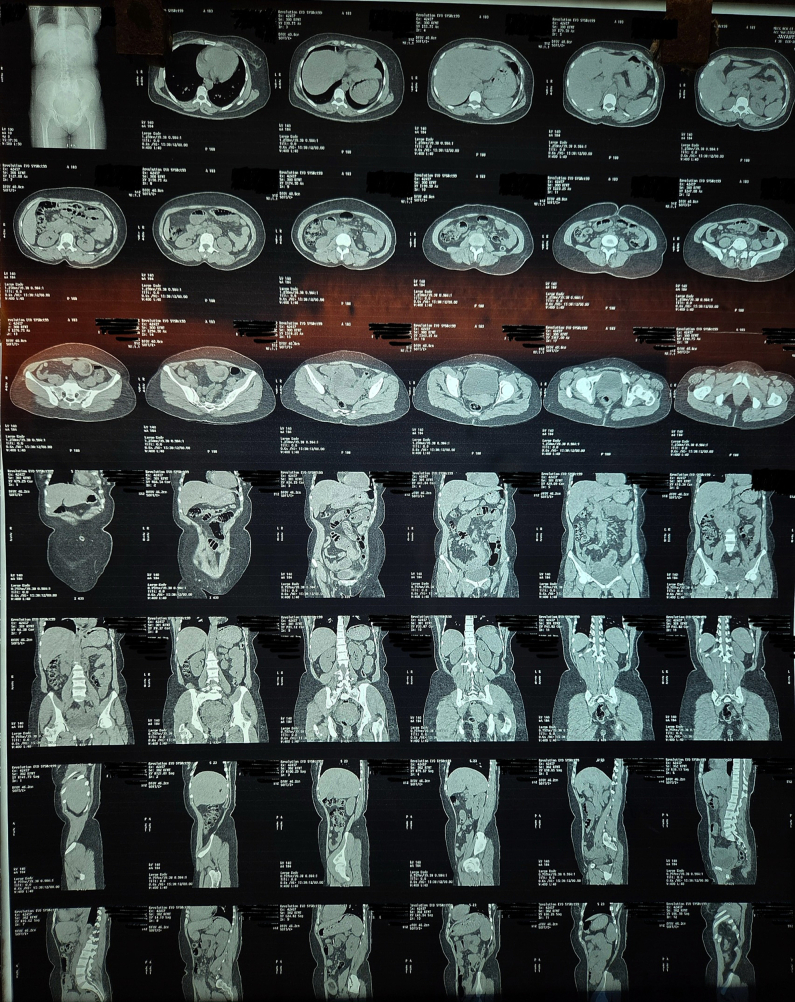
Non Contrast Computed Tomography of Abdomen and Pelvis (Zoomed Out).

**Fig. 2:**
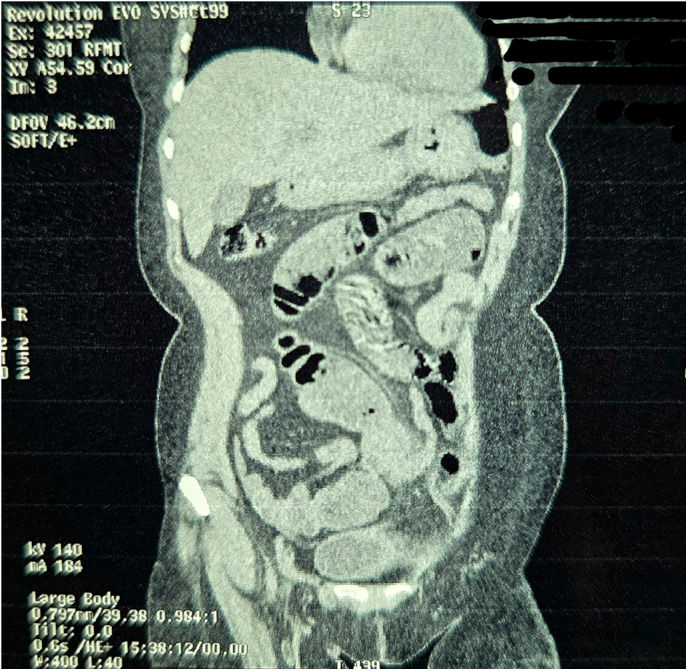
Non Contrast Computed Tomography of Abdomen and Pelvis (Coronal Section Zoomed In).

**Fig. 3:**
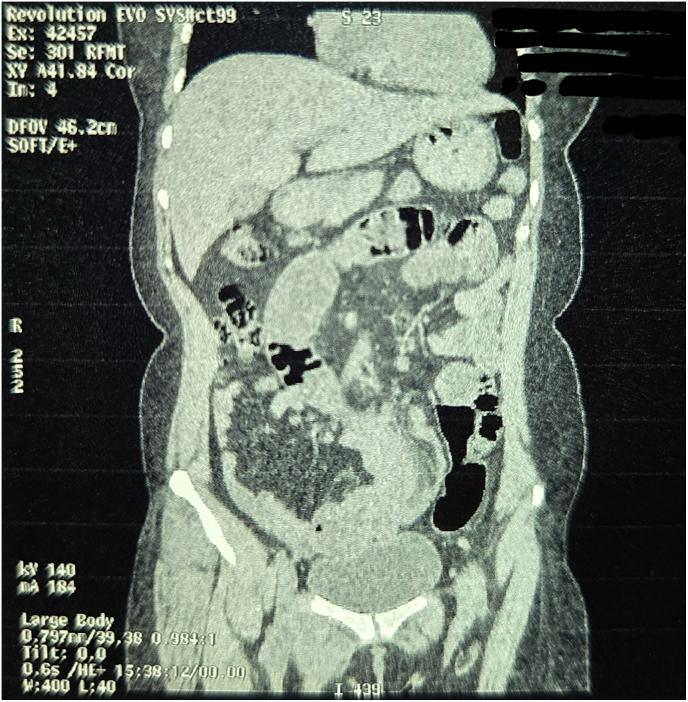
Non Contrast Computed Tomography of Abdomen and Pelvis (Coronal Section Zoomed In).

**Fig. 4:**
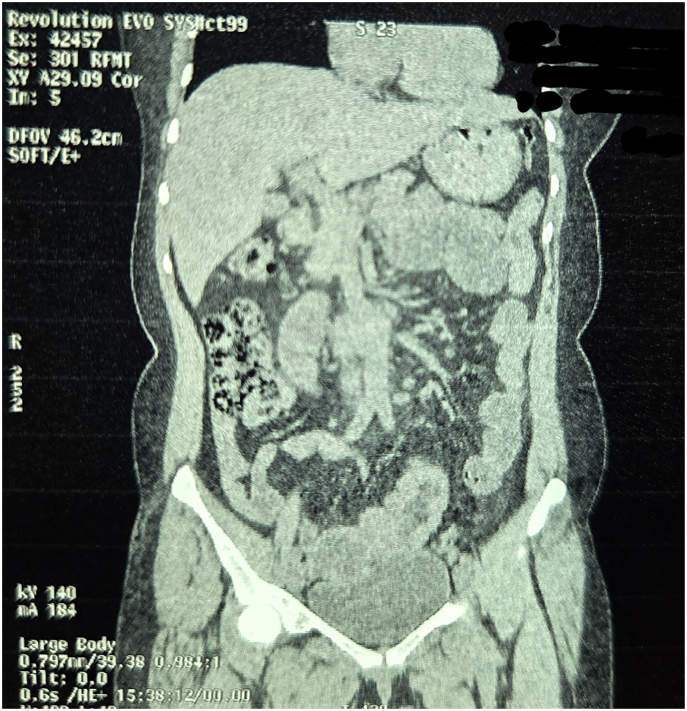
Non Contrast Computed Tomography of Abdomen and Pelvis (Coronal Section Zoomed In).

**Fig. 5:**
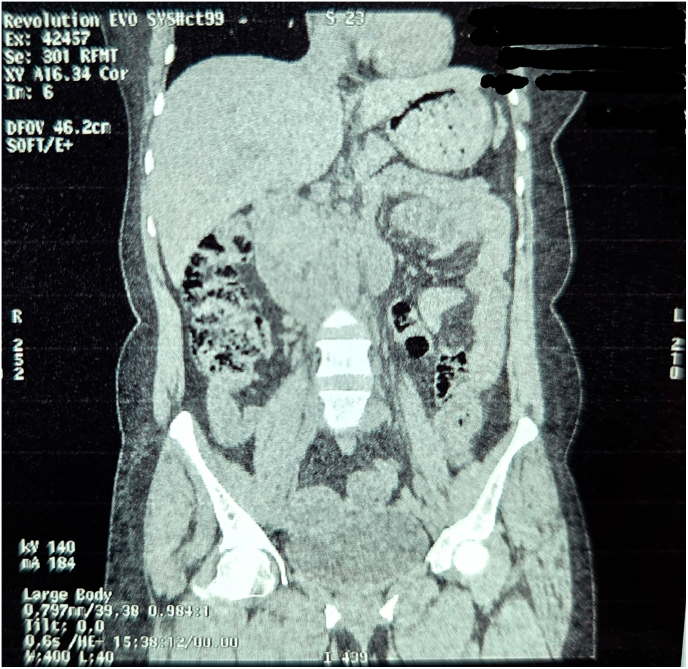
Non Contrast Computed Tomography of Abdomen and Pelvis (Coronal Section Zoomed In).

**Fig. 6:**
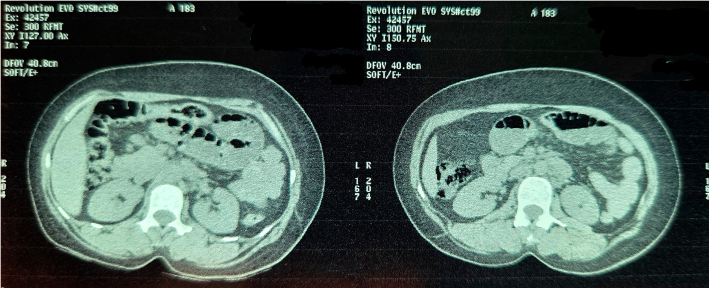
Non Contrast Computed Tomography of Abdomen and Pelvis (Transverse Section Zoomed In).

**Fig. 7:**
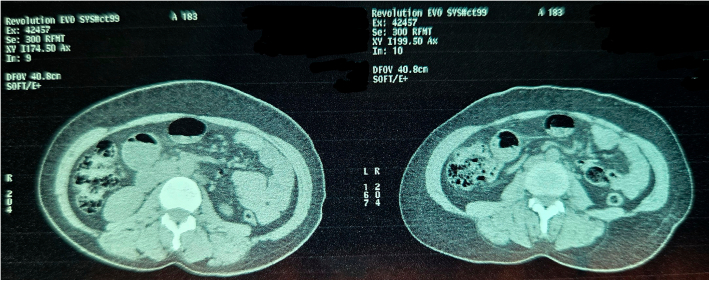
Non Contrast Computed Tomography of Abdomen and Pelvis (Transverse Section Zoomed In).

**Fig. 8:**
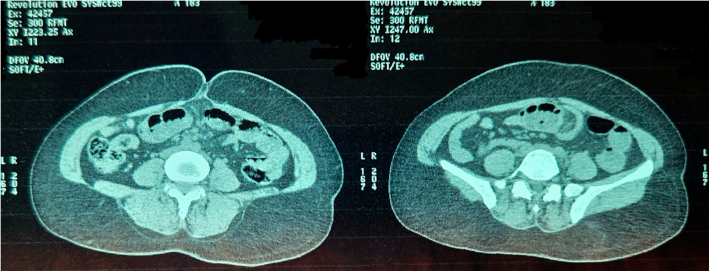
Non Contrast Computed Tomography of Abdomen and Pelvis (Transverse Section Zoomed In).

**Fig. 9:**
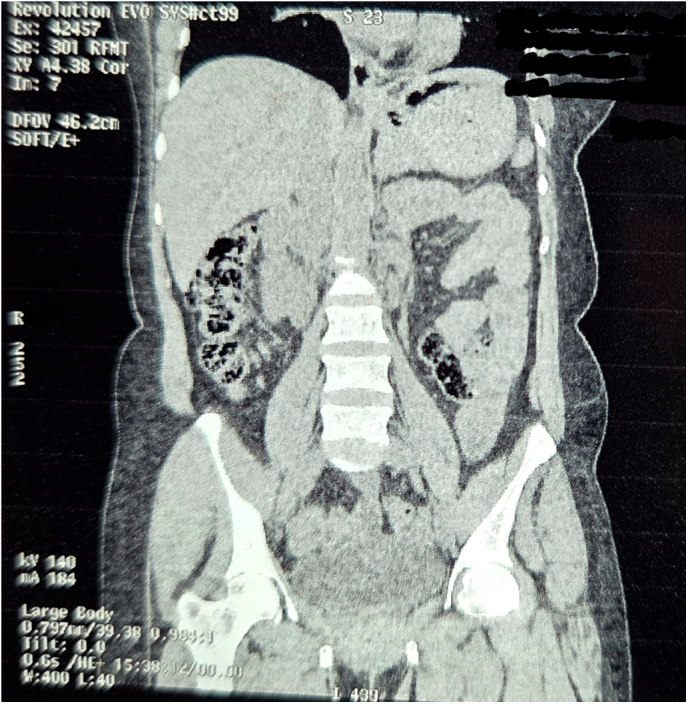
Non Contrast Computed Tomography of Abdomen and Pelvis (Coronal Section Zoomed In).

**Fig. 10:**
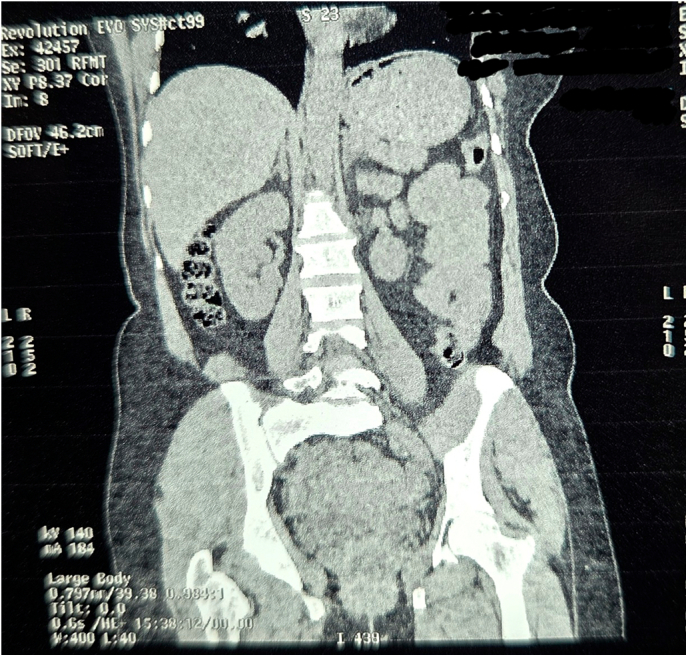
Non Contrast Computed Tomography of Abdomen and Pelvis (Coronal Section Zoomed In).

**Fig. 11:**
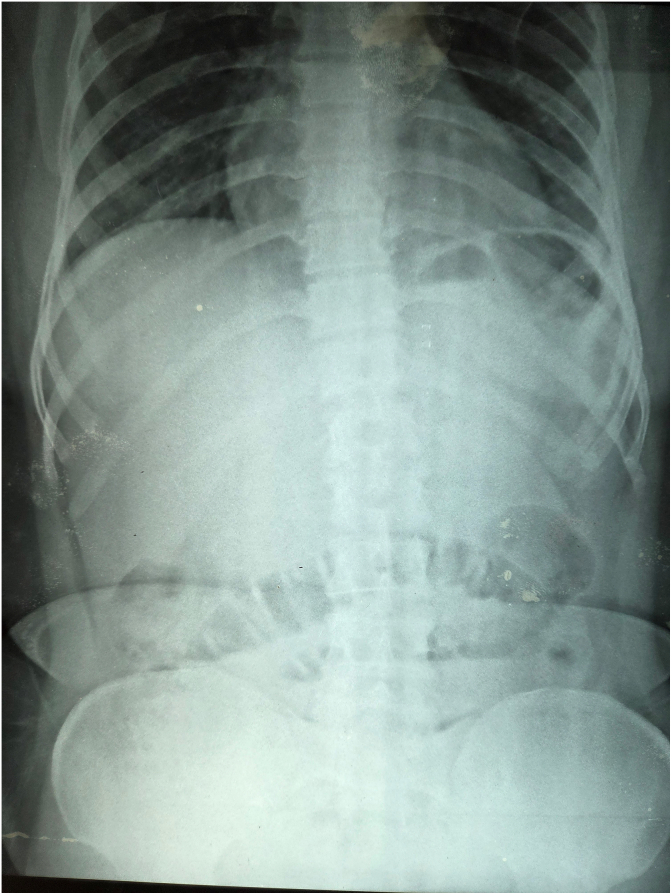
Digital X Ray Abdomen and Pelvis.

Given the CT findings and clinical features of obstruction, the patient was taken for emergency exploratory laparotomy. The decision for surgery was based on the radiological identification of intussusception with a suspected lead point, clinical signs of bowel compromise, and the risk of ischemia or perforation if delayed. The goal was to treat the patient, relieve the acute intestinal obstruction, resect the affected segment, and prevent further complications (Fig. 11).

### 2.2 Surgical findings

Emergency laparotomy revealed a mid-ileum segment telescoped into the adjacent distal segment, forming an intussusception(Fig. 16, Fig. 17, Fig. 18). This segment had further invaginated into a more distal ileal segment, forming double ileo-ileal intussusception. Both intussusceptions were irreducible and showed early ischemia. A 30 cm segment of ileum was resected, and a primary end-to-end anastomosis was performed.

### 2.3 Histopathology

**Fig. 12:**
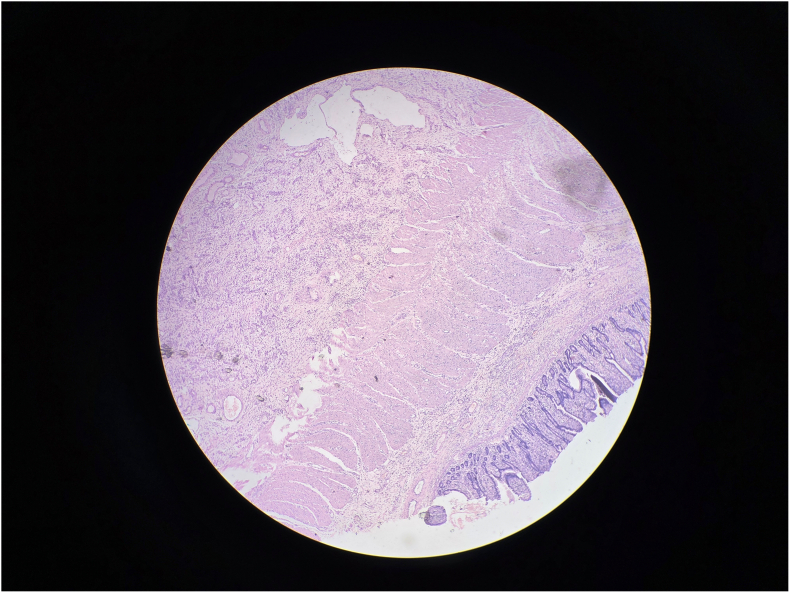
Histopathology Slide (Zoomed Out).

**Fig. 13:**
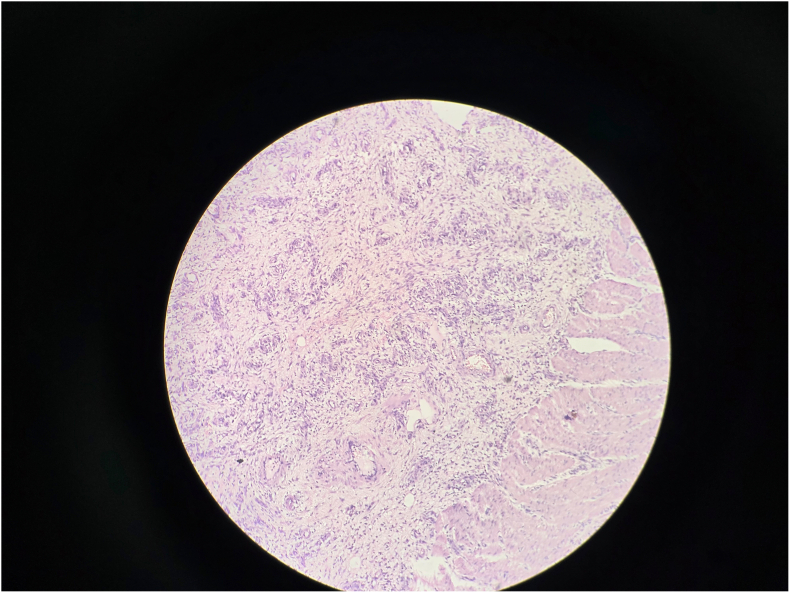
Histopathology Slide (Zoomed In).

**Fig. 14:**
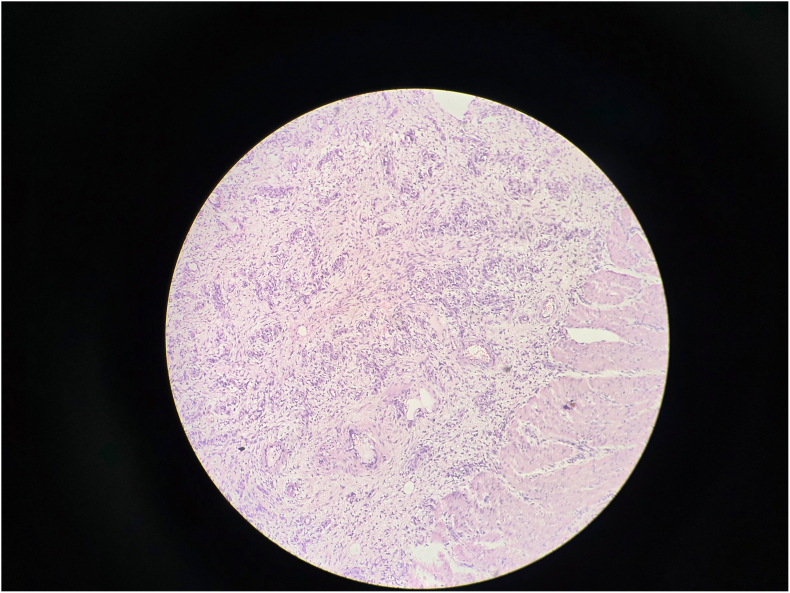
Histopathology Slide (Zoomed In).

**Fig. 15:**
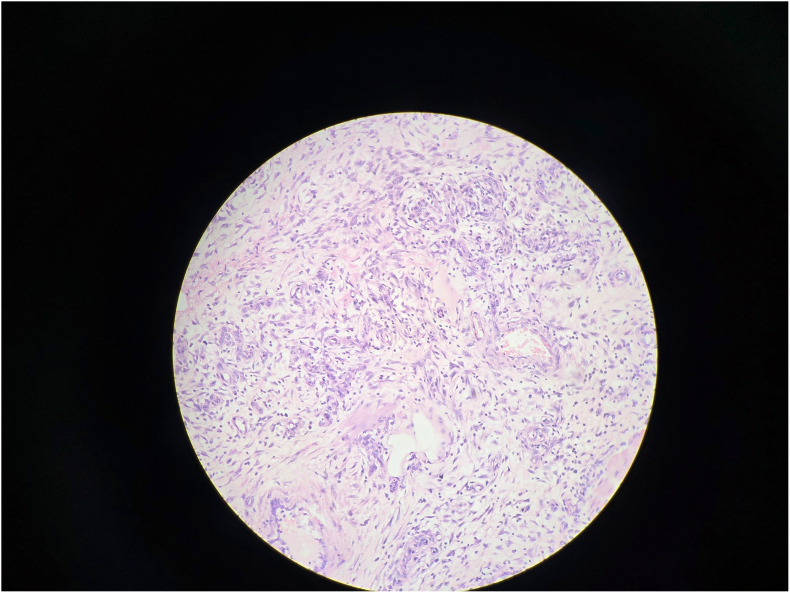
Histopathology Slide (Zoomed In).

Gross examination revealed a 3.5 cm intraluminal polypoid mass at the apex of the inner intussusceptum(Fig. 18). Microscopic examination showed spindle cell proliferation with dense inflammatory infiltrate, including lymphocytes and plasma cells(Fig. 12, Fig. 13, Fig. 14, Fig. 15). Immunohistochemistry confirmed inflammatory pseudotumor (Vimentin positive, Smooth muscle actin positive, Anaplastic lymphoma kinase 1 negative, CD117 negative).

**Fig. 16:**
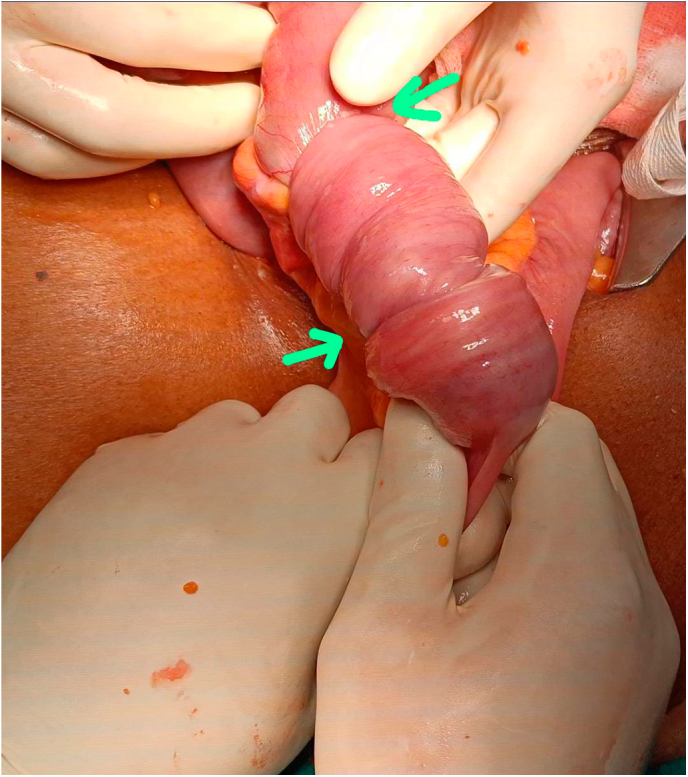
Intra Operative Finding of Double Ileo-ileal Intussusception.

**Fig. 17:**
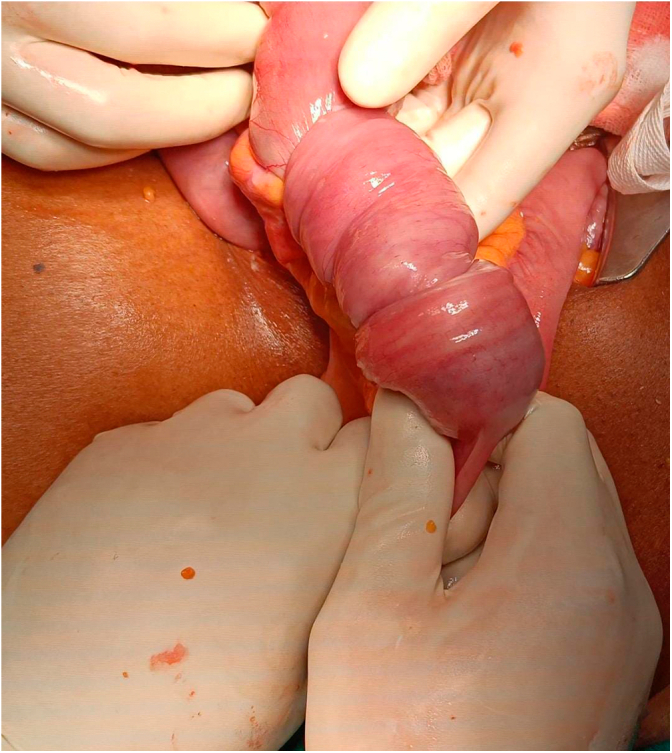
Intra Operative Finding of Double Ileo-ileal Intussusception.

**Fig. 18:**
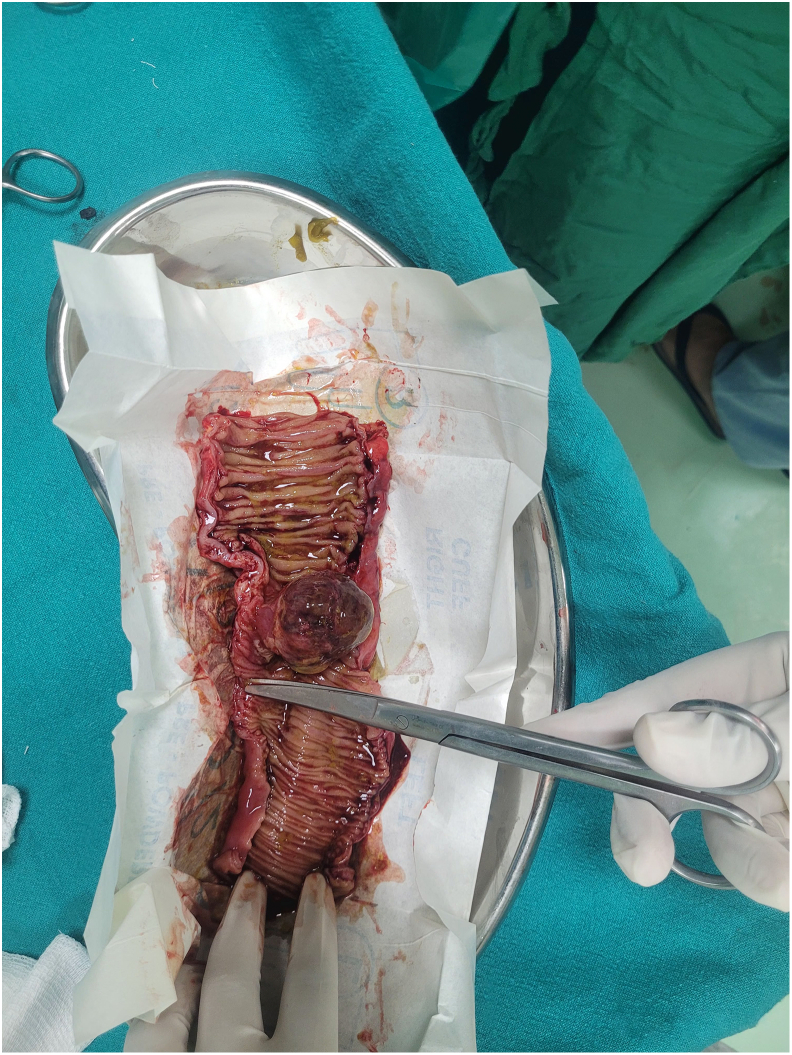
Intraoperative Finding of Intraluminal Mass which acted as the Lead Point.

The procedure was performed by a board-certified consultant general surgeon with over 10 years of experience in gastrointestinal surgery. A 30 cm segment of affected ileum was resected en bloc. No deviations from the planned surgical approach were necessary, and the operation proceeded smoothly.

Postoperative care included nil per os (NPO) for 48 h, intravenous fluids, analgesia, and continued antibiotics for 5 days. The patient's postoperative course was uncomplicated, and she experienced no major adverse events. The surgical outcome can be classified as Clavien-Dindo Grade I. The patient was discharged on postoperative day 7 with normal bowel function. She was followed up at 2 months with no recurrence, and abdominal ultrasonography showed no residual or recurrent lesion.

Timeline of events

**Table:** 

Time	Event
Day 1	Patient presents to emergency department with abdominal pain, vomiting, distension, and absence of bowel movements.
Day 1	Laboratory tests and non-contrast CT scan performed, revealing ileo-ileal intussusception.
Day 1	Emergency laparotomy performed with resection of 30 cm of affected ileum and anastomosis.
Postoperative Day 1–2	Postoperative care with intravenous fluids, antiemetics, and antibiotics.
Postoperative Day 7	Discharged with normal bowel function and no complications.
2-Month Follow-Up	No recurrence observed, and abdominal ultrasonography showed no residual or recurrent lesion.

## 3 Discussion

Adult intussusception is a rare condition that accounts for only 1–5 % of all cases of intestinal obstruction. Unlike in children, where the condition is often idiopathic, adult intussusception is typically caused by a structural lesion, such as neoplasms, Meckel's diverticulum, or postoperative adhesions [1,2]. Inflammatory pseudotumor, a rare benign tumor, is an infrequent but recognized cause of adult intussusception, particularly in the small intestine. Inflammatory pseudotumors are composed of inflammatory cells and fibroblasts and can lead to bowel obstruction by acting as a lead point [3,4]. It is characterized by a myofibroblastic cell proliferation accompanied by a chronic inflammatory infiltrate comprising lymphocytes, plasma cells, and histiocytes. The etiology is uncertain but is postulated to involve an exaggerated immune response to infection, trauma, or autoimmune processes. While inflammatory pseudotumors may mimic malignancies both clinically and radiologically, they typically lack features such as high mitotic activity, significant nuclear atypia, or invasive growth on histology. They are more commonly seen in children and young adults, but the potential for these lesions to cause intussusception in adults should not be overlooked [5,6].

Double intussusception, in which one intussuscepted segment becomes the lead point for another, is exceedingly rare. A handful of cases have been reported in the literature, typically associated with benign conditions such as inflammatory pseudotumors [8]. The pathophysiology behind this phenomenon suggests that increased peristaltic activity and segmental mobility of the bowel may allow a previously intussuscepted segment to serve as the lead point for further telescoping. This results in a complex form of intussusception that is often difficult to diagnose preoperatively and presents significant surgical challenges [9].

In the case presented here, the adult double ileo-ileal intussusception caused by an inflammatory pseudotumor was initially suspected based on imaging findings. However, the double configuration was only confirmed intraoperatively, which highlights the importance of maintaining a high index of suspicion when clinical symptoms suggest obstruction. Computed tomography (CT) is the imaging modality of choice for diagnosing adult intussusception, but complex forms, such as double intussusception, may not always be fully appreciated on imaging alone [10,11]. This underscores the need for prompt surgical exploration when clinical signs point to potential ischemia or obstruction.

Surgical resection without prior reduction is generally recommended in adult intussusception, particularly when malignancy is suspected or when imaging is inconclusive. This approach minimizes the risk of intraluminal dissemination and ensures that any pathological lead point is removed in its entirety [12,13]. In the current case, surgical resection was performed, and the histopathological examination confirmed the diagnosis of inflammatory pseudotumor, which is typically benign but can exhibit locally aggressive behavior [5,14,15]. Immunohistochemistry supported the diagnosis with positive staining for vimentin and smooth muscle actin, and negative markers for ALK-1 and CD117. Although inflammatory pseudotumors are not considered malignant, long-term follow-up is advised due to their unpredictable biological behavior and potential for recurrence [14,15].

In reviewing the literature, it is apparent that while there have been a few documented cases of adult double intussusception due to benign lesions, the occurrence of such cases remains extremely rare. This report adds to the body of knowledge on the condition and highlights the need for a high index of suspicion and timely surgical intervention in similar cases. Further studies are needed to establish more definitive management protocols for such complex cases of adult intussusception.

### 3.1 Strengths and limitations

A key strength of this case is the documentation of an exceedingly rare Double Intussusception configuration due to Inflammatory Pseudotumor in an adult, contributing valuable insight to the limited literature on this subject. Intraoperative findings were carefully described and histologically confirmed, enhancing the report's reliability.

Limitations include the absence of intraoperative photographs due to logistical constraints and the lack of long-term follow-up beyond six months. Additionally, while no complications were encountered, the generalizability of management strategies in this case should be cautiously considered, given its rarity.

### 3.2 Clinical implications and hypothesis generation

This case reinforces the need for early surgical intervention in adult intussusception with suspected lead points, especially when imaging is equivocal. It also highlights the possibility that benign lesions such as Inflammatory Pseudotumor may behave in ways that mimic malignant or complex anatomical disorders. The presentation of Double Intussusception should be added to the differential diagnosis when radiological findings are ambiguous or inconsistent with clinical severity.

Although this case does not introduce a new surgical technique or device, it underscores the importance of individualized surgical decision-making in rare presentations. Reporting such cases contributes to hypothesis generation regarding the pathophysiology of Double intussusception and the behavior of Inflammatory Pseudotumors in adult patients.

This case was not associated with any novel or adverse reactions to surgical devices or drugs, and therefore did not necessitate reporting to any national regulatory agencies.

### 3.3 Conclusion and key takeaways

This report details a rare instance of Double ileo-ileal intussusception secondary to an inflammatory pseudotumor in an adult. The case emphasizes:

• The importance of considering benign causes such as Inflammatory Pseudotumor in adult intussusception,
• The diagnostic limitations of imaging in complex anatomical scenarios,
• And the value of early surgical exploration and en bloc resection for definitive management.

Complete excision was curative, and the patient remains well on follow-up. Surgeons and radiologists should be aware of such rare anatomical patterns, as timely recognition and intervention are essential to prevent complications.

## Authors' contributions

All authors contributed equally to the conception, design, case management, patient care, literature review, manuscript drafting, critical revision, and final approval of the manuscript.

## Consent

Yes, written informed consent was obtained from the patient for publication of this case report and any accompanying images. A copy of the signed consent form is available for review by the journal editor upon request.

## Ethical approval

Ethical approval was not required for this case report as it involved an emergency surgical procedure performed as part of routine clinical care, and it did not involve a first-in-man (FIM) intervention or investigational protocol. Written informed consent for publication was obtained from the patient.

## Guarantor

One of the authors is the guarantor of this manuscript and accepts full responsibility for the work.

## Patient perspective

The patient expressed gratitude for the prompt diagnosis and surgical intervention. She reported relief from symptoms shortly after the procedure and was satisfied with the information provided by the clinical team throughout her care. At her six-month follow-up, she described feeling well, having returned to her normal daily activities, and appreciated the explanation of her condition and the rarity of her case. She expressed support for sharing her experience to help inform and educate others.

## Research registration number

Not applicable.

## Registration of research studies

This case report does not describe a ‘First in Man’ study; therefore, trial registration is not required in accordance with the guidelines of the International Journal of Surgery Case Reports.

## Funding

This research did not receive any specific grant from funding agencies in the public, commercial, or not-for-profit sectors.

## Declaration of competing interest

The authors declare that there are no conflicts of interest regarding the publication of this case report.
